# Topics and Sentiment Surrounding Vaping on Twitter and Reddit During the 2019 e-Cigarette and Vaping Use–Associated Lung Injury Outbreak: Comparative Study

**DOI:** 10.2196/39460

**Published:** 2022-12-13

**Authors:** Dezhi Wu, Erin Kasson, Avineet Kumar Singh, Yang Ren, Nina Kaiser, Ming Huang, Patricia A Cavazos-Rehg

**Affiliations:** 1 Department of Integrated Information Technology University of South Carolina Columbia, SC United States; 2 Department of Psychiatry Washington University School of Medicine St. Louis, MO United States; 3 Department of Artificial Intelligence and Informatics Mayo Clinic Rochester, MN United States

**Keywords:** vaping, e-cigarette, social media, Twitter, Reddit, e-cigarette and vaping use–associated lung injury, EVALI, sentiment analysis, topic analysis

## Abstract

**Background:**

Vaping or e-cigarette use has become dramatically more popular in the United States in recent years. e-Cigarette and vaping use–associated lung injury (EVALI) cases caused an increase in hospitalizations and deaths in 2019, and many instances were later linked to unregulated products. Previous literature has leveraged social media data for surveillance of health topics. Individuals are willing to share mental health experiences and other personal stories on social media platforms where they feel a sense of community, reduced stigma, and empowerment.

**Objective:**

This study aimed to compare vaping-related content on 2 popular social media platforms (ie, Twitter and Reddit) to explore the context surrounding vaping during the 2019 EVALI outbreak and to support the feasibility of using data from both social platforms to develop in-depth and intelligent vaping detection models on social media.

**Methods:**

Data were extracted from both Twitter (316,620 tweets) and Reddit (17,320 posts) from July 2019 to September 2019 at the peak of the EVALI crisis. High-throughput computational analyses (sentiment analysis and topic analysis) were conducted. In addition, in-depth manual content analyses were performed and compared with computational analyses of content on both platforms (577 tweets and 613 posts).

**Results:**

Vaping-related posts and unique users on Twitter and Reddit increased from July 2019 to September 2019, with the average post per user increasing from 1.68 to 1.81 on Twitter and 1.19 to 1.21 on Reddit. Computational analyses found the number of positive sentiment posts to be higher on Reddit (*P*<.001, 95% CI 0.4305-0.4475) and the number of negative posts to be higher on Twitter (*P*<.001, 95% CI –0.4289 to −0.4111). These results were consistent with the clinical content analyses results indicating that negative sentiment posts were higher on Twitter (273/577, 47.3%) than Reddit (184/613, 30%). Furthermore, topics prevalent on both platforms by keywords and based on manual post reviews included mentions of youth, marketing or regulation, marijuana, and interest in quitting.

**Conclusions:**

Post content and trending topics overlapped on both Twitter and Reddit during the EVALI period in 2019. However, crucial differences in user type and content keywords were also found, including more frequent mentions of health-related keywords on Twitter and more negative health outcomes from vaping mentioned on both Reddit and Twitter. Use of both computational and clinical content analyses is critical to not only identify signals of public health trends among vaping-related social media content but also to provide context for vaping risks and behaviors. By leveraging the strengths of both Twitter and Reddit as publicly available data sources, this research may provide technical and clinical insights to inform automatic detection of social media users who are vaping and may benefit from digital intervention and proactive outreach strategies on these platforms.

## Introduction

### Background

In the United States, vaping has become dramatically more popular in recent years, with 1 in every 20 American adults using vaping devices and >2 million middle- and high-school students in the United States using e-cigarettes in 2021 [1,2]. Vaping places individuals at risk for several negative health consequences including diminished lung function and cardiac performance, susceptibility to nicotine dependence, and impacted neurological development, particularly among youth [3,4]. However, despite these negative health consequences, youth and young adults have been found to report limited understanding of the dangers of vaping [5,6] and high perceived ability to quit vaping if desired [7]. Of further concern, e-cigarette and vaping use–associated lung injury (EVALI) resulted in hospitalizations and deaths in 2019, and many of these cases were later linked to vitamin E acetate (a filler substance in unregulated products) [8]. In the context of these risks and negative health outcomes, the United States Food and Drug Administration–labeled vaping among teens as a national epidemic in 2018 and continues to release policies to regulate vaping products more effectively [9]. Given the deleterious health effects of vaping and increased risks for EVALI, future research on publicly available, larger-scale data from sources such as social media are necessary to monitor this growing public health concern and to inform outreach interventions for vaping cessation. Previous literature has leveraged social media data for surveillance of health topics, including illicit drug use [10], mental well-being [11,12], public health [13,14], and other health-related experiences [15]. Twitter is a social media site that is used by approximately 22% (1/5) of American adults [16] as a source of information as well as information sharing [17]. Individuals on web-based platforms such as Twitter may be more willing to openly share experiences and personal stories about mental health or substance misuse with reduced fears of judgment or legal action, allowing them to access social support and advice and share this advice with others who are going through similar experiences [18]. For example, a study of 1200 tweets during mental health awareness week found that awareness, stigma, and personal experiences were central themes of discourse among Twitter users [19]. As such, Twitter has been used as a mass data source of information for public health monitoring and can be used to better understand attitudes and behaviors of individuals in relation to vaping [20-22]. For instance, during the COVID-19 pandemic, Twitter data were used to better understand sentiment and reactions to smoking in relation to the virus [23] as well as individual perspective of global-scale events and US-related lifestyle changes [24]. Although Twitter has several strengths related to surveillance and public health monitoring, other social media platforms such as Reddit may have complementary strengths to provide data on individual-level user vaping behaviors.

Reddit is a similar pseudonymous social media platform used by the public to discuss personal experiences that may be stigmatizing [25-27], including young adults who may disclose personal information with less fear of offline harm or consequences [28]. Reddit data have been used to investigate attitudes and behaviors of individuals who use illicit substances [29-31], and similar research has been conducted among those who vape. One analysis of Reddit threads indicated that primary motivations for vaping among individuals with mental illness include self-medication, freedom and control, vaping as a hobby, social connectedness, as well as vaping to quit smoking [32]. Other studies have used Reddit data to analyze public responses and concerns about vape bans [33], communities supporting e-cigarette cessation [34], and attitudes and reviews toward e-cigarette products [35].

Both Twitter and Reddit are popular social media platforms, but they differ in multiple ways that impact users’ posting behaviors and post content. Twitter, with >300 million monthly active users [36], only allows short 280-character tweets for breaking news, trends, and opinions, often leading to incomplete or misleading statements [37]. In contrast, Reddit, with >430 million actively monthly users, has no character posting limit, is anonymous, and comprises network of communities, namely, subreddits, dedicated to specific topics, allowing users to relate to other individuals with similar backgrounds, views, and lived experiences. With Reddit’s anonymity, people can honestly voice their own opinions with in-depth text and content to spread awareness and important news [38,39]. Thus, posts about the same topic during the same period (ie, posts about vaping in 2019) are expected to vary with regard to the type of content shared and the level of impact on public perception based on the platform on which they are shared.

Large-scale evaluations using computer science (CS) strategies, including those using natural language processing and machine learning for text mining, have been conducted previously on vaping content from social media [40-42]. For example, Visweswaran et al [41] developed machine learning classifiers to identify vaping-relevant tweets toward the development of a vaping surveillance system. Results demonstrate that social media content can be used for overall infoveillance, and such data could inform future, individual-level detection models to identify at-risk posts and users. A systematic review conducted by Kwon and Park [32] found that sentiment regarding vaping tended to be more positive across social media sites, and previous research on Twitter has demonstrated that those who smoke are more likely to engage with misinformation about vaping [43]. Studies conducted on Reddit posts have illustrated health symptoms associated with vaping [44] and highlighted communities aimed to support those wanting to quit vaping [45].

### Objectives

Studying the EVALI public health crisis specifically could aid in the identification of content and keywords related to both acute and long-term health outcomes associated with vaping shared on social media, as such signals of vaping risk may have been amplified during this period. By leveraging the strengths of both Twitter and Reddit as publicly available data sources as well as using an interdisciplinary approach to analyze complex social media content, technical and clinical insights may be garnered to inform the future development of an automatic detection model to connect with vaping users who may benefit from digital intervention on social media platforms. However, to date, there are few studies comparing insights from both Twitter and Reddit for substance misuse within the same time frame [46,47], and no known studies related to vaping have been conducted to analyze the 2019 EVALI outbreak at both the individual user level, and population level. As such, this paper examined vaping-related content on Twitter and Reddit to better understand the (1) sentiment and keywords associated with vaping-related content during the 2019 EVALI time frame, (2) differences in sentiment and keywords between content on Twitter and Reddit, and (3) similarities or differences between statistical analyses and clinical coding of vaping-related content.

## Methods

### Data Collection

In this study, we focused on comparing vaping-related keyword frequencies and sentiment on Twitter and Reddit during the EVALI outbreak period using data from both platforms from July 2019 to September 2019, as our previous work had identified this as a time frame during which vaping-related social media content increased [48]. To define the criteria for large-scale data extraction, our team first conducted a manual analysis of 200 randomly selected vaping-related tweets across the 2019 time line to generate a list of clinically relevant keywords. Our primary research questions guided the creation of this keyword list, which included *vaping*, *vape*, and 60 other specifying terms (Multimedia Appendix 1). Using this set of keywords, a random sample of 316,620 vaping-related tweets with an average of 27 words per tweet was extracted during the EVALI outbreak period (July, August, and September 2019). For comparison purposes, we used the same set of keywords to randomly extract Reddit data, resulting in 17,320 Reddit posts with an average of 211 words per post associated with vaping during the EVALI outbreak.

GetOldTweets [49] is an open-source python library that allowed our team to extract a random sample of tweets with our identified vaping keywords. This module permitted access to and extraction of historical tweets of any date and topic. The benefit of using this application program interface (API) is that it had no restrictions on size and provided access to historical tweets [49,50]. We used Pushshift Reddit API (version 4.0) [51], which provided rich features for searching and extraction and flexible ways to aggregate publicly available Reddit posts and comments.

### Data Cleaning

After we extracted posts from Twitter and Reddit based on the keywords, we cleaned our data sets before further analysis. As we only focused on English-language posts in this study, we first removed the posts that contain non-English languages. We also removed invalid Reddit posts marked as “removed” or “deleted.” After that, the number of Twitter posts reduced from 316,620 to 286,703, and the number of Reddit posts reduced from 17,320 to 12,069.

For the text in the posts, we first converted all the characters to lowercases to avoid the case-sensitive process. Then, we removed all special characters non–American Standard Code for Information Interchange from the text. For text contractions, we expanded them into multiple individual words. Next, we removed the stop words that have no significant contributions to the meaning of the text from the text (eg, is, a, the, and of). After that, we removed the special terms from the tweet text, including mentions, hashtags, links, ticks, punctuations, numbers, and over spaces. Then, we applied the word lemmatization function to convert the words to their base forms.

### Sentiment Analysis

Sentiment analysis is a common computer technique to measure the subjectivity, opinions, attitudes, and emotions in texts [52]. Sentiment analysis quantifies the sentiment contents in a given text along a continuum scale, for example, from −1 to 1 [41,53]. We applied Valence Aware Dictionary and sEntiment Reasoner (VADER) as the tool to analyze the sentiment of tweets and Reddit posts, as VADER is a lexicon and rule-based sentiment analysis tool [54] that recent studies [24,55,56] have found to effectively calculate sentiment social media analysis. More specifically, VADER has been attuned to social media sentiments and pretrained by a gold standard sentiment lexicon, which was developed based on mature sentiment word-banks, popular sentiment expression, and common slang with sentiment value in social media. To determine the sentiment, VADER maps lexical features to emotion intensities known as a sentiment score, which can be obtained by summing up the intensity of each word in the text. The score is then normalized to −1 (most extreme negative) and +1 (most extreme positive). In our study, if the text sentiment score was >0, then the text was classified as positive. The text was classified as negative if the sentiment score was <0. The neutral text’s sentiment score was 0. Our study further classified posts into positive, negative, and neutral sentiment toward vaping using this sentiment score, calculating the distribution of the posts in terms of the 3 sentiment types per month.

### Keyword Analysis

In addition to the sentiment analysis described earlier, we used chi-square tests to compare differences between the frequency of keywords in Twitter and Reddit posts during each month across the following topics: (1) sentiment, (2) emotion-related keywords, (3) health-related keywords, (4) age-related keywords, (5) marketing-related keywords, (6) product-related keywords, (7) addiction-related keywords, and (8) quitting-related keywords.

### Term Frequency–Inverse Document Frequency

Term frequency–inverse document frequency (TF-IDF) is a statistical measurement that can represent the word relevant in a corpus [57]. The TF-IDF score is calculated based on the term frequency and inverse document frequency. Using this method helps us find the common words on Twitter and on Reddit. On the basis of the TF-IDF scores, we can identify the most important words on both the platforms. The formulas are as follows:

TF = number of a word in the document / number of words in the document **(1)**

IDF = log(number of documents / number of documents with the word) **(2)**

TF-IDF = TF × IDF **(3)**

### Clinical Coding Comparison

During the EVALI outbreak, July, August, and September 2019 were identified as months during and just before the dramatic increase in vaping-related discussions on Twitter based on both the content and sentiment analyses outlined earlier. As such, a random sample of 200 posts per month from the Twitter and Reddit data sets described earlier were extracted for in-depth human coding toward contextual content analysis. Specifically, members of our clinical team with experience in substance use research (students in psychology, social work, or public health at the graduate level and with relevant experience coding qualitative social media data led by author PCR, a clinical psychologist) used inductive and deductive methods to construct a codebook based on a review of sample tweets and informed by previous literature [58,59]. Three primary coding categories were used: (1) type of post, including personal, marketing, or media or news or other [60]; (2) sentiment toward vaping [61]; and (3) health outcomes mentioned, including both positive (eg, quitting combustible smoking) and negative (eg, lung injury, death, and addiction or dependence) [60,62]. Secondary concepts that were coded as either present or not present included (1) mentions teens or adolescents or young adults [63] and (2) mentions marijuana or weed or cannabidiol or tetrahydrocannabinol [64,65]. Two independent human coders reviewed each post and assigned applicable codes based on text content, and agreement among coders was substantial as reflected by an average κ score of 0.62 [66]. A third coder then reviewed the coding from each preliminary coder and provided final codes for those tweets on which there was disagreement [67], which is a third-party resolution method used in previous qualitative analysis literature [68]. Both frequency and qualitative themes were then compared with the preliminary results from the CS analyses to aid in the conceptualization of the clinical themes reflected in the data set.

Total frequency of each theme mentioned on both Twitter and Reddit was compared across the months of July, August, and September 2019 (sum of 3 months) to demonstrate relative weight of each topic on the respective platforms.

### Ethics Approval

The Washington University Institutional Review Board (202101009) reviewed the methods of data extraction and analysis for this study. Given that the data are publicly available on social media, the study was determined to be nonhuman subjects research and exempt from review.

## Results

### Data Set Summary and Unique Users

This section presents the results from the high-throughput computational analyses. In total, we collected 286,703 tweets and 12,096 posts on Reddit. The sample size differences between Twitter and Reddit were related to the amount of information included in each Reddit post and in a tweet. The word limit for each tweet is 280 characters, whereas the word limit for each Reddit post is 40,000 characters. Thus, each Reddit post included much richer information than a tweet. To analyze the data set at the word level and further content analysis, the number of extracted Reddit posts was significantly smaller than the number of tweets. Table 1 presents the number of unique users and posts per user on both platforms. Overall, the number of vaping-related posts and unique users on Twitter and Reddit had an increasing trend from July 2019 to September 2019. In particular, the number of posts and unique users on Twitter increased by approximately 4 times from August 2019 to September 2019. The number of posts per user on Twitter and Reddit increased from 1.68 to 1.81 and 1.19 to 1.21, respectively.

**Table 1:** Number of unique users and posts per user on Twitter and Reddit mentioning vaping during the e-cigarette and vaping use–associated lung injury outbreak.

Month in 2019	Unique users, n (%)		Posts per user, n	
	Twitter	Reddit	Twitter	Reddit
July	17,904 (11.06)	2893 (28.75)	1.68	1.19
August	28,604 (17.67)	3066 (30.47)	1.66	1.2
September	115,373 (71.27)	4105 (40.79)	1.81	1.21

### Sentiment Analysis Results

CS pattern analysis of sentiment found that overall posts with positive sentiment about vaping were more common than negative posts on Reddit (8905/12,096, 73.62%), and negative sentiment was dominant on Twitter (174,448/286,703, 60.86%) during the EVALI period (Table 2). Clinical results based on a small random sample during this period were similar to the results using CS methods, still demonstrating that Reddit had a higher number of positive sentiment posts and also reflecting that Twitter had a higher number of negative sentiment posts based on manual review of post content.

The results of monthly sentiment trends indicated that the percentage of posts with positive sentiment was higher than that with negative sentiment in July both on Twitter and on Reddit. In August and September, the percentage of negative posts was higher than that of the positive ones on Twitter. Moreover, there was a significant decrease in the percentage of positive sentiment from July to September on Twitter, whereas positive posts were dominant on Reddit in August and September.

The chi-square tests (Table S1 in Multimedia Appendix 2) found an overall significant difference in sentiment between platforms. Twitter contained significantly more negative postings (174,488/286,703, 60.86%) than Reddit (2281/12,096, 18.86%), and Reddit contained significantly more positive posts (8905/12,095, 73.62%) than Twitter (85,209/286,703, 29.72%).

In addition to the sentiment analysis and trends, we also ran chi-square tests to compare emotion expression–related posting differences on Twitter and Reddit. We selected common emotional words from the list of most frequent words on both Twitter and Reddit. Positive keywords included *safe, good,* and *love*, and negative keywords included *kill, bad, dangerous, concern,* and *serious*. The statistical results indicated significant posting differences between the 2 platforms as a whole, based on their frequency percentages. We found that positive emotion expressions were much more significant on Reddit than on Twitter in all 3 months during the EVALI outbreak period (Table S2 in Multimedia Appendix 2).

**Table 2:** Sentiment analysis and clinical coding on Twitter and Reddit.

	Sentiment analysis, n (%)			Clinical coding, n (%)	
	Twitter (n=286,703)	Reddit (n=12,096)	Twitter (n=577)		Reddit (n=613)
Positive	85,209 (29.72)	8905 (73.62)	201 (34.8)		291 (47.5)
Negative	174,488 (60.86)	2281 (18.86)	273 (47.3)		184 (30)
Neutral	27,006 (9.42)	910 (7.52)	103 (17.9)		138 (22.5)

### Keyword Analysis by Topic

#### Health-Related Keyword Analysis

**Figure 1:**
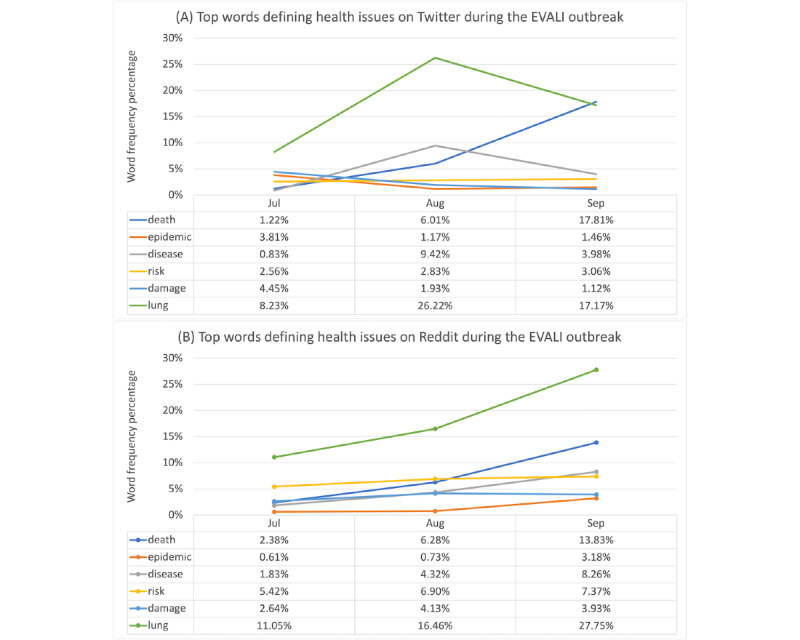
Top words defining health issues on Twitter (A) and Reddit (B) during the e-cigarette and vaping use–associated lung injury (EVALI) outbreak.

The distributions and percentages of the posts that contained vaping health-related keywords are shown in Table 3. Figure 1 presents the frequency of the top 6 words associated with health issues in July, August, and September 2019. The top 6 words were commonly shared between Twitter and Reddit. On the basis of the TF-IDF scores as shown in Multimedia Appendix 3, we found that the most important health-related keywords often mentioned on Twitter included *death, lung, quit, smoking, disease,* and *harm,* whereas the most important words in the Reddit posts included *death, lung, quit, smoking, cough,* and *doctor*.

We performed a chi-square test to compare health-related keywords, including *death, lung, disease, risk, crisis, sick, doctor, cancer, injury, epidemic, research, damage, harm, harmful, patient, cough, chest, prevention, smoking,* and *quit* based on the posts in July, August, and September 2019. The chi-square test results (Table S3 in Multimedia Appendix 2) showed significant differences between health-related keywords posting on Twitter and Reddit for each of the 3 months and as a whole. However, owing to the significant differences between the size of posts on Twitter and Reddit, the overall effect size was small. On the basis of the percentages, more health-related keywords were discussed on Twitter than on Reddit, and negative health outcomes were highly discussed on both Reddit and Twitter.

In addition to investigating the sentiment of health-related keywords, chi-square tests associated with addiction-related keywords (Table S4 in Multimedia Appendix 2) showed significant differences and small effect sizes between platforms in each month and the entire EVALI outbreak period. On the basis of percentages, the addiction-related keywords were mentioned more significantly on Twitter than on Reddit.

Within the in-depth clinical coding, negative health outcomes were mentioned much more frequently on both the platforms (Twitter: 230/577, 39.9% and Reddit: 227/578, 39.3%) than positive health outcomes (Twitter: 134/577, 23.2% and Reddit: 182/578, 31.5%). Additional topic mentioned within these negative health outcomes included EVALI/hospitalization, which was more prevalent on Twitter (Twitter: 176/577, 30.5% and Reddit: 146/578, 25.3%), whereas addiction or dependence on vaping products was mentioned more often on Reddit (Twitter: 57/577, 9.9% and Reddit: 123/578, 21.3%). Those mentioning positive health outcomes related to vaping were more common on Reddit, consistent with the keyword analysis described earlier; further, clinical coding found that vaping as a means of quitting combustible smoking was more often mentioned on Reddit than on Twitter (Twitter: 118/577, 20.5% and Reddit: 177/578, 30.6%).

**Table 3:** Distribution and percentage of health-related keywords on Twitter and Reddit.

Health-related keywords	July 2019, n (%)			August 2019, n (%)			September 2019, n (%)			Total, n (%)	
	Twitter	Reddit	Twitter		Reddit	Twitter		Reddit	Twitter		Reddit
death	343 (1.1)	70 (2)	2701 (5.67)		129 (3.5)	32,971 (15.77)		493 (9.9)	36,015 (12.56)		692 (5.7)
lung	2305 (7.67)	281 (8.2)	11,612 (24.39)		411 (11.2)	33,394 (15.98)		870 (17.5)	47,311 (16.5)		1562 (12.91)
disease	235 (0.8)	56 (2)	4268 (8.96)		112 (3.0)	7969 (3.8)		297 (6.0)	12,472 (4.35)		465 (3.8)
risk	708 (2.4)	147 (4.3)	1231 (2.59)		197 (5.4)	6012 (2.88)		280 (5.6)	7951 (2.77)		624 (5.2)
crisis	81 (0.3)	9 (0.3)	216 (0.5)		10 (0.3)	6072 (2.90)		102 (2.1)	6369 (2.22)		121 (1)
sick	415 (1.4)	148 (4.3)	1054 (2.21)		182 (4.9)	5467 (2.62)		370 (7.5)	6936 (2.42)		700 (5.8)
doctor	682 (2.3)	183 (5.3)	2359 (4.95)		228 (6.2)	4623 (2.21)		323 (6.5)	7664 (2.67)		734 (6.1)
cancer	442 (1.5)	84 (2)	782 (1.64)		80 (2)	3691 (1.77)		139 (2.8)	4915 (1.71)		303 (2.5)
injury	96 (0.3)	31 (1)	1256 (2.64)		45 (1)	3990 (1.91)		104 (2.1)	5342 (1.86)		180 (1.5)
epidemic	1091 (3.63)	16 (0.5)	533 (1.12)		24 (0.7)	2920 (1.40)		139 (2.8)	4544 (1.58)		179 (1.5)
research	523 (1.7)	159 (4.6)	712 (1.49)		178 (4.8)	3006 (1.44)		264 (5.3)	4241 (1.48)		601 (5.0)
damage	1315 (4.37)	78 (2)	882 (1.85)		119 (3.2)	2237 (1.07)		156 (3.1)	4434 (1.55)		353 (2.9)
harm	1503 (5.00)	139 (4.0)	1888 (3.97)		180 (4.9)	8253 (3.95)		303 (6.1)	11,644 (4.06)		622 (5.1)
harmful	492 (1.6)	40 (1)	643 (1.35)		49 (1)	2892 (1.38)		143 (2.9)	4027 (1.40)		232 (1.9)
patient	162 (0.5)	61 (2)	1103 (2.31)		82 (2)	1741 (0.83)		122 (2.5)	3006 (1.05)		265 (2.2)
cough	262 (0.9)	141 (4.1)	415 (0.9)		163 (4.4)	1130 (0.54)		257 (5.2)	1807 (0.63)		561 (4.6)
chest	105 (0.4)	108 (3.1)	127 (0.3)		133 (3.6)	431 (0.2)		227 (4.6)	663 (0.2)		468 (3.9)
prevention	96 (0.3)	6 (0.2)	246 (0.5)		14 (0.4)	529 (0.3)		31 (1)	871 (0.3)		51 (0.4)
smoking	3486 (11.60)	430 (12.5)	4145 (8.71)		435 (11.8)	15,604 (7.47)		604 (12.2)	23,235 (8.10)		1469 (12.15)
quit	3017 (1036)	823 (23.9)	3567 (7.49)		853 (23.2)	17,365 (8.31)		1160 (23.36)	23,949 (8.35)		2836 (23.45)

#### Age-Related Keyword Analysis

**Figure 2:**
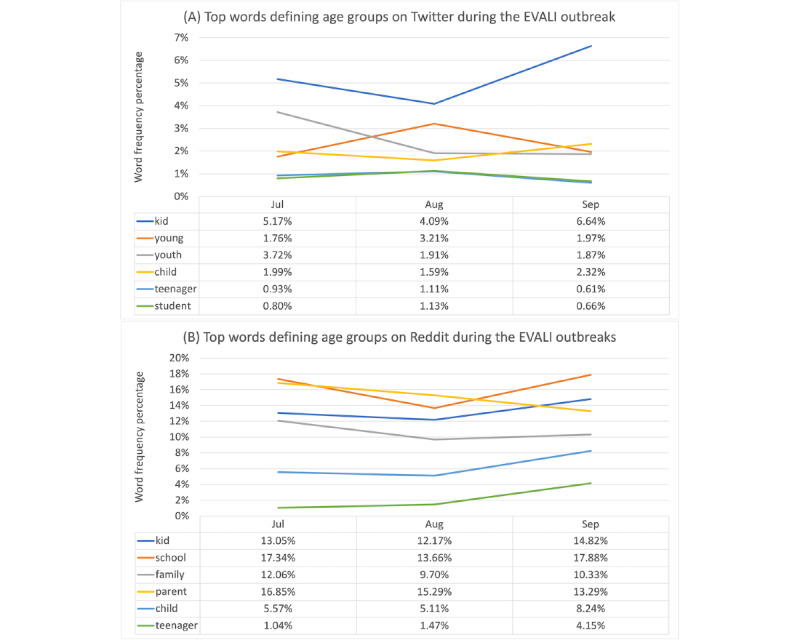
Top words on age groups on Twitter (A) and Reddit (B) during the e-cigarette and vaping use–associated lung injury (EVALI) outbreak.

The top 6 words related to age groups in July, August, and September 2019 are presented in Figure 2 for Twitter and Reddit based on frequency. Among the age-related keywords, *kids* was the most used word on Twitter and Reddit after August 2019. Other frequently used words on Twitter included *youth, young, child,* and *teenager*. Reddit posts more often contained words such as *parent, school,* and *family*.

Age-related keywords in our data set included *kid, adult, child, young, old, youth, parent, school, age, student, family, teenager, minor, mother, husband, wife, adolescent, father,* and *aunt* in July, August, and September 2019 separately. The chi-square test results (Table S5 in Multimedia Appendix 2) showed significant differences and small effect sizes between age-related keywords on Twitter and Reddit for each of the 3 months and as a whole and indicated that age-related keywords were more frequently mentioned on Twitter than on Reddit. Clinical review of post content focused only on mentions of youth and young adults and found differing results, showing that Twitter had 22.9% (132/577) of tweets mentioning youth and Reddit had 28.5% (165/578) of posts mentioning this group.

#### Marketing-Related Keyword Analysis

**Figure 3:**
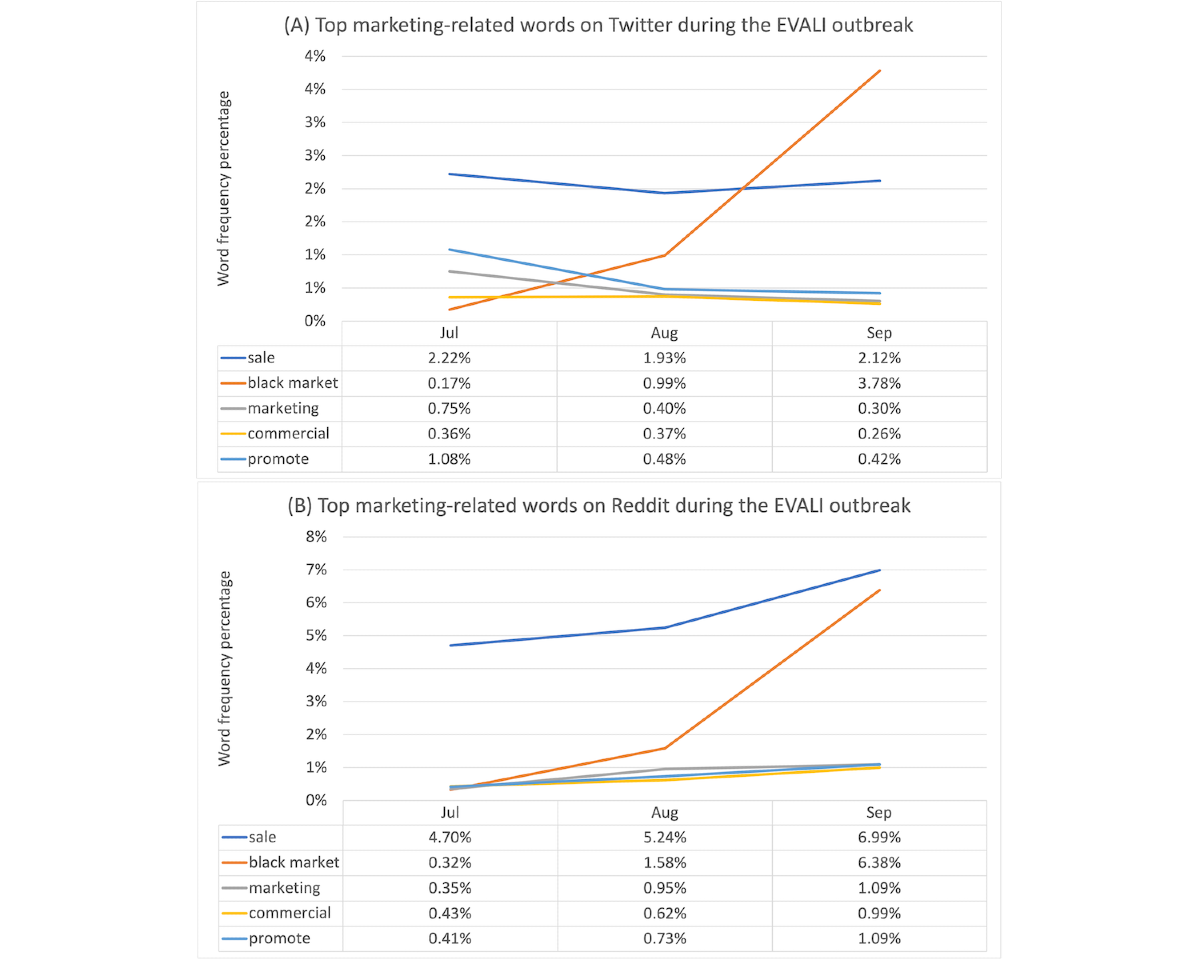
Top marketing-related words on Twitter (A) and Reddit (B) during the e-cigarette and vaping use–associated lung injury (EVALI) outbreak.

Frequently used words about vaping marketing were highly similar between Twitter and Reddit, including *sale, commercial, market, black market,* and *promote*. The trends of top 5 marketing-related words on Twitter and Reddit during the 3 months of EVALI outbreak are illustrated in Figure 3. Mentions of *sale, black market,* and *commercial* increased on Twitter and Reddit from July 2019 to September 2019.

Marketing-related keywords in our data set included *black market, black market, market, sale, news, promote, marketing, commercial, blackmarket,* and *media* for July, August, and September 2019. The chi-square test results (Table S6 in Multimedia Appendix 2) showed significant differences with small effect sizes between marketing-related keywords posting on Twitter and Reddit for all the 3 months, indicating that they were discussed more frequently on Twitter than on Reddit. Clinical analyses focused on marketing regulation and policies and had different results, showing that Twitter discussed policies 8.3% (48/577) of the time and Reddit discussed policies 20% (116/578) of the time.

#### Vaping Product Keyword Analysis

**Figure 4:**
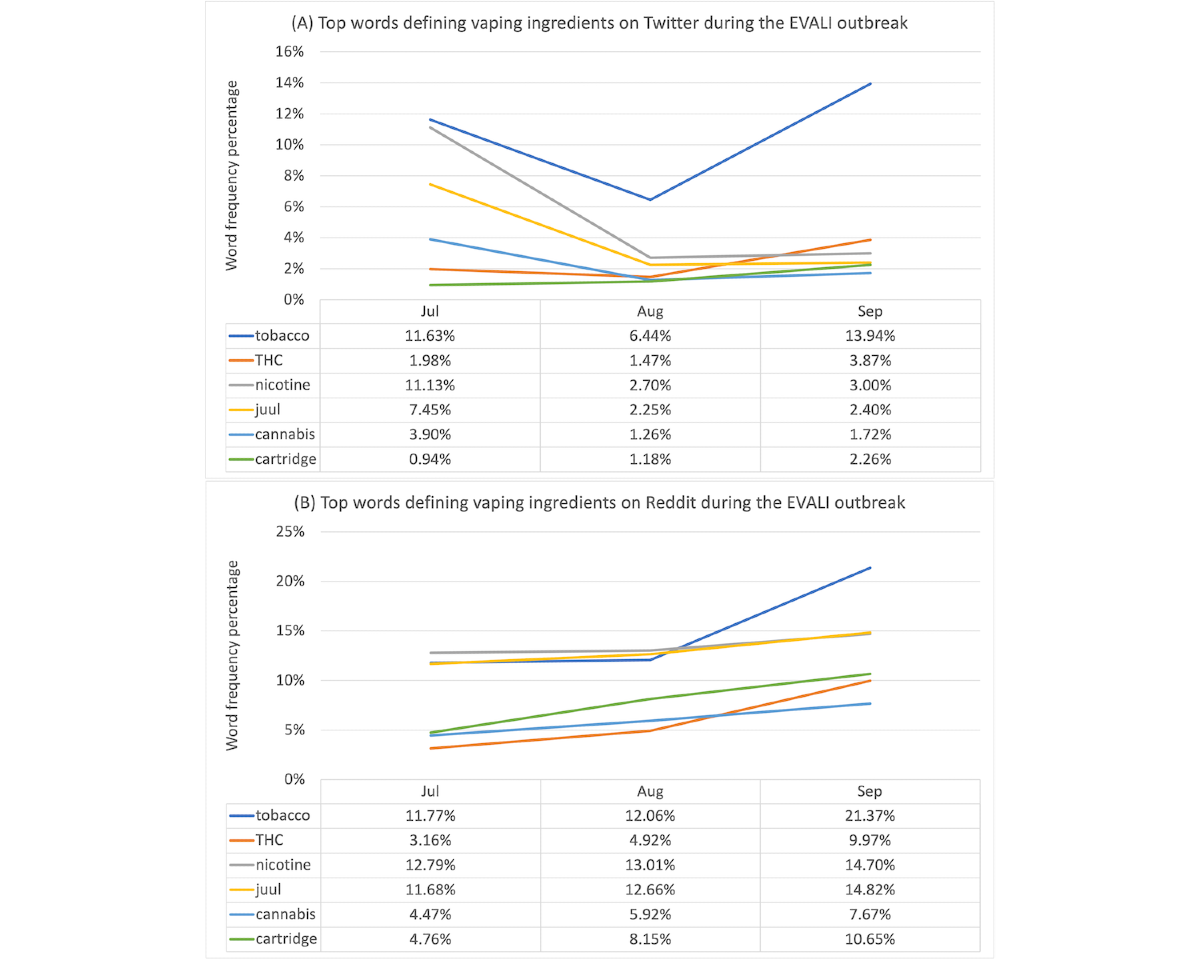
Top words defining vaping ingredients on Twitter (A) and Reddit (B) during the e-cigarette and vaping use–associated lung injury (EVALI) outbreak. THC: tetrahydrocannabinol.

The detailed distributions and percentages of the vaping product keywords are listed in Multimedia Appendix 3, and the top words related to vaping substances on Twitter and Reddit are illustrated in Figure 4. On both platforms, the most frequent word about vaping ingredients or products was *cigarette*, and mentions of marijuana-related keywords (*weed*, *CBD*, *THC*, and *cannabis*) and alcohol were also prevalent. On Reddit, specific keywords about product components, such as *juice, cartridge,* and *liquid*, were slightly more common. The most common words on Reddit included *cigarette*, *product*, and *juice*, which varied across months. The most common words on Twitter included *cigarette*, *tobacco*, and *product* and stayed consistent across August 2019 to September 2019. On the basis of the TF-IDF scores as shown in Multimedia Appendix 3, we found that the most important words in the posts from Twitter included *cig, cigarette, tobacco, product, thc,* and *nicotine,* whereas the most important words in the Reddit posts included *nicotine, cigarette, juice,* and *weed.*Vaping product-related keywords in our data set included *cigarette, tobacco, product, thc, cig, nicotine, juice, juul, cartridge, liquid, cannabis, chemical, alcohol, ecigarette, weed, cbd, flavour,* and *ingredient* based on the data sets in July, August, and September 2019. The chi-square test results (Table S7 in Multimedia Appendix 2) showed significant differences with small effect sizes between vaping product-related keywords posting on Twitter and Reddit for all the 3 months, finding that more vaping product-related keywords were mentioned on Twitter based on percentages. Clinical analyses found different results, showing that marijuana-related keywords were mentioned more than twice as often on Reddit (208/578, 35.9%) than on Twitter (77/577, 13.3%).

#### Quitting Vaping

In addition, quit-related keywords in our data set included *quit*, *quitting, stop,* and *stopper* to compare pattern differences on Twitter and Reddit. The chi-square test results indicated significant posting differences with small effect sizes between the 2 platforms for all the 3 months and as a whole, showing quit-related words mentioned on Twitter more often based on percentages (Table S8 in Multimedia Appendix 2). Clinical analyses showed different results, with Reddit having 21.8% (126/578) of posts related to quitting and Twitter having 6.4% (37/577) of tweets related to quitting.

## Discussion

### Principal Findings and Implications

As vaping has become more popular in recent years, so have discussions about its direction, policies, and health connotations on social media platforms, and this study illustrated differences in sentiment and keyword content on Twitter and Reddit during the EVALI outbreak in 2019. According to the trends in the frequency of vaping-related posts during this time frame, vaping-related content increased slowly between July and August, with a dramatic spike from August to September. Moreover, there was a significant increase in the number of unique Twitter and Reddit users who participated in these discussions during the EVALI outbreak. The fact that increasing trends in the frequency of social media vaping-related content peaked in parallel with the EVALI outbreak and across both popular social media platforms supported the utility of social media as a surveillance system for exploring naturally occurring, real-time reactions and communications during a public health vaping-associated crisis.

Importantly and based on our content analysis, Twitter and Reddit content within posts about vaping were found to contain primarily positive sentiment about vaping. However, the 2 platforms were notably different based on the most prevalent type of content identified. Specifically, Reddit users tended to reveal personal vaping experiences and opinions about vaping benefits, policies, and products, including how potential restrictive vaping policies may have negative impacts on users who vape (ie, less access to vaping products that aid cigarette smoking cessation). Mentions of marijuana were also >2 times as high on Reddit as on Twitter and often included queries to other Reddit users about the safety of specific vaping products and which symptoms, if any, should warrant concern or medical care. In contrast, Twitter included more mainstream media content surrounding vaping, specifically related to the rise in EVALI cases. We also observed that Twitter feeds contained attention-grabbing negative sentiment and higher use of negative emotional expressions, including *kill*, *bad,* *dangerous*, *concern*, and *serious,* as well as increased content on possible negative health outcomes of vaping, including addiction. Although both platforms had mentions of youth, Twitter highlighted headlines about the youth vaping epidemic and EVALI among teens and ways to limit vaping products for adults who use them as smoking cessation aids, whereas on Reddit, mentions related to youth mostly were individuals describing their own vaping behaviors, including initiating vaping behaviors as a teen.

In summary, we observed numerous and meaningful distinctions in the frequencies of content topics across both social media outlets. These differences may be owing to the way individuals socially network as well as their motive for discussion on each platform. For instance, information on Twitter is known as “the” social media platform for news coverage, and it is most often used by journalists and major news providers to broadcast news and update the public in real time as important events transpire [69]. This may explain why Twitter had a higher frequency of negative posts related to vaping, as journalists and their audience leveraged this platform for updates and interactions throughout the unfolding of the EVALI outbreak, especially as it evolved into a crisis that resulted in many hospitalizations and several deaths. In contrast, Reddit distinguishes itself from other social media platforms by facilitating more candid discussions, including exchanges about substance use behaviors, given its pseudonymous user system and generous character limit restrictions; this may be why we found a higher prevalence of content describing one’s personal experiences with vaping.

### Comparison With Previous Work

It might also be that the differences we found were owing to the distinctions between the users themselves. For instance, the demographic user base of Twitter is predominantly White adults, who have a higher degree of education and are more likely to be identified as Democrat than the general public, with 10% of users creating 80% of the tweets [70]. In contrast, Reddit users tend to span degrees of education attainment and live in urban or suburban areas [71]. The Centers for Disease Control and Prevention finds that within people of color, there are higher percentages of individuals who vape compared with the percentage of White people who vape [72], and another study shows that higher level of education attainment was linked to lower odds of e-cigarette use [73]. This suggests that users on Reddit may be more likely to vape than users on Twitter, explaining their different sharing patterns of personal vaping-related experiences and concerns over restrictive policy.

### Limitations

The findings of this paper should be considered within the context of its limitations. First, we analyzed only text-based posts or messages on these platforms. Although this provided us with data-heavy information from each social media site, it did not include the multitude of multimedia content including photos, videos, and links that are available for further analysis. Second, owing to the character limits on Twitter and the unlimited length of Reddit posts, the differences between the number of words in each post could have impacted both the sentiment and keyword analyses in this study. Third, because of the timing of our data gathering, we did not garner information related to COVID-19 and its implications on those who vape and vaping policies, leaving us unable to discern more recent implications. Fourth, our original keyword list used to extract the vaping-related data sets from Twitter and Reddit may have contained more negative health-related keywords, and this could have impacted the results with regard to sentiment and health outcomes, causing a potential selection bias in our keyword list. As this study focused on the health issue regarding vaping-related topics on Twitter and Reddit during the outbreak period, the keyword list included multiple sentiment-related words. It will bring bias to our sentiment analysis results, but these words were the key to selecting the related posts and addressing our research questions. In addition, we applied the GetOldTweets and Pushshift APIs to extract the data based on the keyword list. As the extraction mechanism of these APIs is to find the posts with the same field as one of the keywords without further filtration to matched posts, the extracted data set might include the posts from bots instead of real users, which may introduce bias to our sentiment results. We plan to apply different methods to clean the posts generated by bots in our future studies. However, the use of this data set was in line with the larger aims of this study, which were to better understand the content and sentiment surrounding vaping on Twitter and Reddit to inform the development of potential identification and outreach methods on social media to those at risk of negative health outcomes to improve public health. The fifth limitation was that we applied an existing tool VADER to analyze the sentiment of the posts, and thus, it could bias to our sentiment analysis results, which are common issues for any sentiment analysis tool owing to the complex dynamics of human expressions, emotions, and contexts. In the future, we will also consider creating a sentiment analysis model optimization with social media posts to overcome the current disadvantages of not effectively identifying sarcastic sentences.

### Future Directions

Overall, the results of this study revealed the strengths of both Twitter and Reddit as publicly available social media data sources as a public health crisis transpired and evolved. Health practitioners working with those who vape or who have interest in quitting vaping should be aware of the information and possible misinformation related to vaping and work to assess whether social media engagement on various platforms could impact continued use or be a barrier to cessation. The results shared in this manuscript could also inform social media companies and public health officials by alerting them to the marketing of vaping products on these sites and encouraging protections for communities such as those on Reddit aimed to support vaping cessation. In addition, to improve public health reach, future research could explore automatic detection mechanisms that leverage each platform’s content and type of networking identified here, especially to study the potential for identifying users that are vaping and may want information and support to quit. This could help lead to efficient and timely social media informed proactive outreach strategies to distribute health education about vaping, including strategies for vaping cessation.

## Appendix

Multimedia Appendix 1 Keywords and terms used for data extraction.

Multimedia Appendix 2 Statistical analysis results.

Multimedia Appendix 3 Distribution and term frequency–inverse document frequency score of vaping product–related keywords on Twitter and Reddit.

## Abbreviations

API: application program interface
CS: computer science
EVALI: e-cigarette and vaping use–associated lung injury
TF-IDF: term frequency–inverse document frequency
VADER: Valence Aware Dictionary and Sentiment Reasoner

## Data Availability

Access to the data set analyzed in this manuscript may be made available to researchers via reasonable request to the corresponding author.
