# Supplementary material for: Topics and Sentiment Surrounding Vaping on Twitter and Reddit During the 2019 e-Cigarette and Vaping Use–Associated Lung Injury Outbreak: Comparative Study
Source: J Med Internet Res. 2022 Dec 13;24(12):e39460. doi: 10.2196/39460 (PMC9795395; doi:10.2196/39460)
Supplement: Multimedia Appendix 1 [file jmir_v24i12e39460_app1.docx]

**Appendix 1: Keywords/Terms Used for Data Extraction**

**Keyword lists used to extract vaping-related tweets on Twitter and posts on Reddit:**

lung, injury, health, illness, popcorn, epidemic, disease, die, dying, breathe, breathing, cough, coughing, trouble, problem, sick, safer, addicted, pneumonia, hospital, healthy, healthcare, ecigs, ecigarette, electronic, vaper, vapor, vapers, quit, quitting, smoke, smoking, safe, better, cigarette, tobacco, lungs, injuries, illnesses, popcornlung, death, deaths, evali, respiratory, fatal, case, cases, crisis, danger, dangers, dangerous, concern, concerns, cause, causes, severe, threatening, doctor, risk, addiction, addictive, addicting.

Above words were combined with the words ‘vape’ and ‘vaping’.
