# Supplementary material for: Topics and Sentiment Surrounding Vaping on Twitter and Reddit During the 2019 e-Cigarette and Vaping Use–Associated Lung Injury Outbreak: Comparative Study
Source: J Med Internet Res. 2022 Dec 13;24(12):e39460. doi: 10.2196/39460 (PMC9795395; doi:10.2196/39460)
Supplement: Multimedia Appendix 2 [file jmir_v24i12e39460_app2.docx]

**Multimedia Appendix 2:**

**Statistical Analysis Results:**

| Sentiment  Analysis | Platform | No. of Posts for the Targeted Sentiment | No. of Posts for Other Sentiments | Chi-Square Value | *P* value | Effect Size (Phi) |
| --- | --- | --- | --- | --- | --- | --- |
| Negative | Twitter | 174,488 (60.86%) | 112,215 | 8474.91 | *P* < .001 | 0.17 |
|  | Reddit | 2,281 (18.86 %) | 9,815 |  |  |  |
| Positive | Twitter | 85,209 (29.72 %) | 201,494 | 10366.3 | *P* < .001 | -0.19 |
|  | Reddit | 8,905 (73.62 %) | 3,191 |  |  |  |
| Neutral | Twitter | 27,006 (9.42 %) | 259,697 | 49.28 | *P* < .001 | 0.01 |
|  | Reddit | 910 (7.52 %) | 11,186 |  |  |  |

Table S1. Chi-Square Testing on Sentiment-Related Keywords

| Platform | Positive Emotion Expression  Keyword Frequency | Negative Emotion Expression  Keyword Frequency | Chi-Square Value | *P* value | Effect Size (Phi) |
| --- | --- | --- | --- | --- | --- |
| Twitter | 36,590 (30.61%) | 82,943 | 579.27 | *P* < .001 | -0.06 |
| Reddit | 11,555 (37.81%) | 19,004 |  |  |  |

Table S2. Chi-Square Testing on Emotion Expression-Related Keyword Postings

| Month | Platform | Health-related Keyword Frequency | Non-Health-related Keyword Frequency | Chi-Square Value | *P* value | Effect Size (Phi) |
| --- | --- | --- | --- | --- | --- | --- |
| July 2019 | Twitter | 18,951 (3.70%) | 493,495 | 10,306.92 | *P* < .001 | 0.10 |
|  | Reddit | 4,363 (0.80%) | 541,406 |  |  |  |
| August 2019 | Twitter | 42,664 (5.60%) | 719,822 | 19,549.66 | *P* < .001 | 0.12 |
|  | Reddit | 5,490 (0.98%) | 553,500 |  |  |  |
| September 2019 | Twitter | 173,894 (4.93%) | 3,351,369 | 17,619.63 | *P* < .001 | 0.06 |
|  | Reddit | 9,457 (1.37%) | 680,981 |  |  |  |
| Total | Twitter | 235,509 (4.91%) | 4,564,686 | 51,615.80 | *P* < .001 | 0.09 |
|  | Reddit | 19,310 (1.08%) | 1,775,887 |  |  |  |

Table S3. Chi-Square Testing on Health-related Keyword Postings

| Month | Platform | Addiction-related Keyword Frequency | Non-Addiction-related Keyword Frequency | Chi-Square Value | *P* value | Effect Size (Phi) |
| --- | --- | --- | --- | --- | --- | --- |
| July 2019 | Twitter | 776 (0.15%) | 511,670 | 426.36 | *P* < .001 | 0.02 |
|  | Reddit | 171 (0.03%) | 545,598 |  |  |  |
| August 2019 | Twitter | 1,127 (0.15%) | 761,359 | 369.53 | *P* < .001 | 0.02 |
|  | Reddit | 222 (0.04%) | 558,768 |  |  |  |
| September 2019 | Twitter | 3,384 (0.10%) | 3,521,879 | 53.02 | *P* < .001 | 0.003 |
|  | Reddit | 463 (0.07%) | 689,975 |  |  |  |
| Total | Twitter | 5,287 (0.11%) | 4,794,908 | 547.75 | *P* < .001 | 0.01 |
|  | Reddit | 856 (0.04%) | 1,794,341 |  |  |  |

Table S4. Chi-Square Testing on Addiction-related Keyword Postings

| Month in 2019 | Platform | Age-related Keyword Frequency | Non-Age-related Keyword Frequency | Chi-Square Value | *P* value | Effect Size (Phi) |
| --- | --- | --- | --- | --- | --- | --- |
| July | Twitter | 12,290 (2.40%) | 500,156 | 1737.62 | *P* < .001 | 0.04 |
|  | Reddit | 7,147 (1.31%) | 538,622 |  |  |  |
| August | Twitter | 15,813 (2.07%) | 746,673 | 1126.12 | *P* < .001 | 0.03 |
|  | Reddit | 7266 (1.30%) | 551724 |  |  |  |
| September | Twitter | 70,991 (2.01%) | 3,454,272 | 771.22 | *P* < .001 | 0.01 |
|  | Reddit | 10,431 (1.51%) | 680,007 |  |  |  |
| Total | Twitter | 99,094 (2.06%) | 4,701,101 | 3281.03 | *P* < .001 | 0.02 |
|  | Reddit | 24,844 (1.38%) | 1,770,353 |  |  |  |

Table S5. Chi-Square Testing Results on Age-related Keywords

| Month | Platform | Market-related Keyword Frequency | Non-Market-related Keyword Frequency | Chi-Square Value | *P* value | Effect Size (Phi) |
| --- | --- | --- | --- | --- | --- | --- |
| July 2019 | Twitter | 2,842 (0.55%) | 509,604 | 1287.32 | *P* < .001 | 0.03 |
|  | Reddit | 797 (0.15%) | 544,972 |  |  |  |
| August 2019 | Twitter | 6,100 (0.80%) | 756,386 | 2081.58 | *P* < .001 | 0.04 |
|  | Reddit | 1,154 (0.21%) | 557,836 |  |  |  |
| September 2019 | Twitter | 47,405 (1.34%) | 3,477,858 | 4152.39 | *P* < .001 | 0.03 |
|  | Reddit | 2,925 (0.42%) | 687,513 |  |  |  |

| Total | Twitter | 56,347 (1.17%) | 4,743,848 | 11565.00 | *P* < .001 | 0.04 |
| --- | --- | --- | --- | --- | --- | --- |

Table S6. Chi-Square Testing Results on Market-related Keyword Postings

| Month | Platform | Vaping Product-related Keyword Frequency | Non-Vaping Product-related Keyword Frequency | Chi-Square Value | *P* value | Effect Size (Phi) |
| --- | --- | --- | --- | --- | --- | --- |
| July 2019 | Twitter | 37,753 (7.37%) | 474,693 | 18242.89 | *P* < .001 | 0.13 |
|  | Reddit | 10,342 (1.89%) | 535,427 |  |  |  |
| August 2019 | Twitter | 49,111 (6.76%) | 677,375 | 15509.98 | *P* < .001 | 0.11 |
|  | Reddit | 11,529 (2.06%) | 547,461 |  |  |  |
| September 2019 | Twitter | 276,881 (7.85%) | 3,248,382 | 23163.59 | *P* < .001 | 0.07 |
|  | Reddit | 18,905 (2.74%) | 671,533 |  |  |  |
| Total | Twitter | 363,745 (7.58%) | 4,436,450 | 63900.68 | *P* < .001 | 0.10 |
|  | Reddit | 40,776 (2.27%) | 1,754,421 |  |  |  |

Table S7. Chi-Square Testing Results on Vaping Product-related Keywords

| Month | Platform | Quit-related Keyword Frequency | Non-Quit-related Keyword Frequency | Chi-Square Value | *P* value | Effect Size (Phi) |
| --- | --- | --- | --- | --- | --- | --- |
| July 2019 | Twitter | 5,003 (0.98%) | 507,443 | 1043.32 | *P* < .001 | 0.03 |
|  | Reddit | 2,459 (0.45%) | 543,310 |  |  |  |
| August 2019 | Twitter | 6,511 (0.85%) | 755,975 | 750.03 | *P* < .001 | 0.02 |
|  | Reddit | 2,549 (0.46%) | 556,441 |  |  |  |
| September 2019 | Twitter | 35,069 (1.00%) | 3,490,194 | 1625.01 | *P* < .001 | 0.02 |
|  | Reddit | 3,386 (0.49%) | 687,052 |  |  |  |
| Total | Twitter | 46,583 (0.97%) | 4,753,612 | 3996.84 | *P* < .001 | 0.02 |
|  | Reddit | 8,394 (0.47%) | 1,786,803 |  |  |  |

Table S8. Chi-Square Testing on Quit-related Keyword Postings
