# Supplementary material for: Topics and Sentiment Surrounding Vaping on Twitter and Reddit During the 2019 e-Cigarette and Vaping Use–Associated Lung Injury Outbreak: Comparative Study
Source: J Med Internet Res. 2022 Dec 13;24(12):e39460. doi: 10.2196/39460 (PMC9795395; doi:10.2196/39460)
Supplement: Multimedia Appendix 3 [file jmir_v24i12e39460_app3.docx]

**Multimedia Appendix 3:** Distribution and TF-IDF score of vaping product–related keywords on Twitter and Reddit

| Health-related keywords | July 2019 | | | | August 2019 | | | | September 2019 | | | | Total | | | |
| --- | --- | --- | --- | --- | --- | --- | --- | --- | --- | --- | --- | --- | --- | --- | --- | --- |
|  | Twitter | | Reddit | | Twitter | | Reddit | | Twitter | | Reddit | | Twitter | | Reddit | |
|  | n  (%) | TF-IDF^a^ | n (%) | TF-IDF | n (%) | TF-IDF | n (%) | TF-IDF | n (%) | TF-IDF | n (%) | TF-IDF | n (%) | TF-IDF | n (%) | TF-IDF |
|  | | | | | | | | | | | | | | | | |
| death | 343 (1.1) | 73.12 | 70 (2) | 5.20 | 2701 (5.67) | 647.27 | 129 (3.5) | 12.48 | 32,971 (15.77) | 5361.30 | 493 (9.9) | 47.85 | 36,015 (12.56) | 6075.68 | 692 (5.7) | 68.69 |
| lung | 2305 (7.67) | 463.93 | 281 (8.2) | 26.94 | 11,612 (24.39) | 1707.25 | 411 (11.2) | 41.89 | 33,394 (15.98) | 5084.23 | 870 (17.5) | 76.08 | 47,311 (16.50) | 7243.41 | 1562 (12.91) | 145.49 |
| disease | 235 (0.8) | 50.73 | 56 (2) | 3.93 | 4268 (8.96) | 895.28 | 112 (3.0) | 12.44 | 7969 (3.8) | 1727.03 | 297 (6.0) | 28.78 | 12,472 (4.35) | 2668.03 | 465 (3.8) | 46.10 |
| risk | 708 (2.4) | 136.24 | 147 (4.3) | 11.02 | 1231 (2.59) | 249.84 | 197 (5.4) | 15.95 | 6012 (2.88) | 1166.31 | 280 (5.6) | 24.28 | 7951  (2.77) | 1545.39 | 624 (5.2) | 50.70 |
| crisis | 81 (0.3) | 22.88 | 9 (0.3) | 0.69 | 216 (0.5) | 59.26 | 10 (0.3) | 0.54 | 6072 (2.90) | 1382.48 | 102 (2.1) | 8.33 | 6369  (2.22) | 1460.62 | 121 (1) | 10.11 |
| sick | 415 (1.4) | 102.37 | 148 (4.3) | 15.91 | 1054 (2.21) | 199.60 | 182 (4.9) | 16.39 | 5467 (2.62) | 957.74 | 370 (7.5) | 31.26 | 6936  (2.42) | 1253.71 | 700 (5.8) | 63.11 |
| doctor | 682 (2.3) | 181.35 | 183 (5.3) | 15.73 | 2359 (4.95) | 560.10 | 228 (6.2) | 21.20 | 4623 (2.21) | 1124.79 | 323 (6.5) | 28.65 | 7664  (2.67) | 1861.24 | 734 (6.1) | 64.79 |
| cancer | 442 (1.5) | 95.16 | 84 (2) | 8.81 | 782 (1.64) | 164.22 | 80  (2) | 8.45 | 3691 (1.77) | 770.40 | 139 (2.8) | 14.87 | 4915  (1.71) | 1025.79 | 303 (2.5) | 31.69 |
| injury | 96 (0.3) | 30.52 | 31 (1) | 1.90 | 1256 (2.64) | 349.64 | 45  (1) | 4.24 | 3990 (1.91) | 995.66 | 104 (2.1) | 12.21 | 5342  (1.86) | 1372.81 | 180 (1.5) | 18.28 |
| epidemic | 1091 (3.63) | 224.08 | 16 (0.5) | 2.94 | 533 (1.12) | 117.03 | 24 (0.7) | 2.52 | 2920 (1.40) | 639.16 | 139 (2.8) | 15.62 | 4544  (1.58) | 976.27 | 179 (1.5) | 21.84 |
| research | 523 (1.7) | 75.5 | 159 (4.6) | 11.78 | 712 (1.49) | 134.99 | 178 (4.8) | 12.55 | 3006 (1.44) | 575.32 | 264 (5.3) | 18.81 | 4241  (1.48) | 783.81 | 601 (5.0) | 42.46 |
| damage | 1315 (4.37) | 339.9 | 78 (2) | 6.38 | 882 (1.85) | 222.34 | 119 (3.2) | 10.14 | 2237 (1.07) | 563.55 | 156 (3.1) | 15.10 | 4434  (1.55) | 1123.79 | 353 (2.9) | 31.30 |
| harm | 1503 (5.00) | 108.53 | 139 (4.0) | 5.23 | 1888 (3.97) | 158.86 | 180 (4.9) | 7.71 | 8253 (3.95) | 603.24 | 303 (6.1) | 10.09 | 11,644 (4.06) | 867.63 | 622 (5.1) | 22.66 |
| harmful | 492 (1.6) | 102.77 | 40 (1) | 5.07 | 643 (1.35) | 144.39 | 49  (1) | 5.07 | 2892 (1.38) | 633.02 | 143 (2.9) | 14.32 | 4027  (1.40) | 877.18 | 232 (1.9) | 24.50 |
| patient | 162 (0.5) | 39.28 | 61 (2) | 3.85 | 1103 (2.31) | 311.75 | 82  (2) | 5.73 | 1741 (0.83) | 441.32 | 122 (2.5) | 12.45 | 3006  (1.05) | 790.35 | 265 (2.2) | 21.73 |
| cough | 262 (0.9) | 71.19 | 141 (4.1) | 18.09 | 415 (0.9) | 108.15 | 163 (4.4) | 21.35 | 1130 (0.54) | 281.77 | 257 (5.2) | 29.43 | 1807  (0.63) | 460.11 | 561 (4.6) | 68.19 |
| chest | 105 (0.4) | 17.4 | 108 (3.1) | 11.53 | 127 (0.3) | 28.13 | 133 (3.6) | 16.54 | 431 (0.2) | 91.55 | 227 (4.6) | 25.11 | 663  (0.2) | 135.57 | 468 (3.9) | 52.94 |
| prevention | 96 (0.3) | 23.7 | 6 (0.2) | 0.54 | 246 (0.5) | 59.88 | 14 (0.4) | 1.32 | 529 (0.3) | 139.81 | 31 (1) | 2.83 | 871  (0.3) | 222.39 | 51 (0.4) | 4.66 |
| smoking | 3486 (11.60) | 498.54 | 430 (12.5) | 36.29 | 4145 (8.71) | 629.49 | 435 (11.8) | 39.16 | 15,604 (7.47) | 2496.36 | 604 (12.2) | 49.20 | 23,235 (8.10) | 3619.40 | 1469 (12.15) | 123.15 |
| quit | 3017 (1036) | 472.85 | 823 (23.9) | 63.25 | 3567 (7.49) | 588.15 | 853 (23.2) | 70.12 | 17,365 (8.31) | 2853.23 | 1160 (23.36) | 93.47 | 23,949 (8.35) | 3908.23 | 2836 (23.45) | 224.51 |

^a^TF-IDF: term frequency–inverse document frequency.

**Table S1.** Distribution and TF-IDF score of vaping product–related keywords on Twitter and Reddit.
